# Supplementary material for: Hand and Foot Selection in Mental Body Rotations Involves Motor-Cognitive Interactions
Source: Brain Sci. 2022 Nov 4;12(11):1500. doi: 10.3390/brainsci12111500 (PMC9688262; doi:10.3390/brainsci12111500)
Supplement: Supplementary file 1 [file brainsci-12-01500-s001.zip › brainsci-1861480-supplementary.pdf]

Hand and foot selection in mental body rotations involves motor-cognitive interactions

Author Information

Stephan F. Dahm [1], Emiko J. Muraki [2], & Penny M. Pexman[2]

[1] UMIT TIROL – private university of health sciences and health technology

[2] University of Calgary

Corresponding author:

Stephan Frederic Dahm

Institute for Psychology

UMIT TIROL – private university of health sciences and health technology

Eduard-Wallnöfer-Zentrum 1

A-6060 Hall in Tyrol

Email: Stephan.Dahm@uibk.ac.at

Tel: +43 (0) 50 8648 – 4031

Overview

This document includes additional analyses that may be of interest to some readers. First, we report separate analyses of response times (RTs) and error rates of Study 2 which are presented in a combined linear speed-accuracy score in the manuscript. Second, correlations between response times and error rates are presented. Third, correlations between LISAS and age / vividness of movement imagery are presented for both studies. Fourth, effects of sex on LISAS in the mental body rotation task were analyzed for both studies. Fifth, leaning effects during familiarization were analyzed in Study 2.

Analysis of response times

A repeated measures ANOVA with the within-subject factors of perspective (front view, back view), rotation (head up, head down), and limb (arm, leg) was calculated on the RTs. Boxplots of the RTs are shown in Figure S1.

The significant main effect of perspective, F (1, 152) = 145.7, p < .001, η_p^2 = .49, indicated significantly longer RTs in the front view (M±SD = 1.9±0.8 s) than the back view (M±SD = 1.6±0.7 s). The significant main effect of rotation, F (1, 152) = 490.5, p < .001, η_p^2 = .76, indicated significantly longer RTs in head down rotations (M±SD = 2.1±0.8 s) than head up rotations (M±SD = 1.3±0.5 s). The significant main effect of limb, F (1, 152) = 96, p < .001, η_p^2 = .39, indicated significantly longer RTs for leg items (M±SD = 1.8±0.8 s) than arm items (M±SD = 1.6±0.7 s). The significant interaction between rotation and limb, F (1, 152) = 13.2, p < .001, η_p^2 = .08, indicated the difference between limbs was significantly larger in head down rotations (Δ M = 0.2 s) than in head up rotations (Δ M = 0.1 s; p < .001, d = 0.3). All remaining interactions were not significant, η_p^2 < .01.

Figure S1. Boxplots of the RTs (in s) depending on rotation (head down, head up), perspective (back view, front view), and limb (arm, leg) in Study 1.

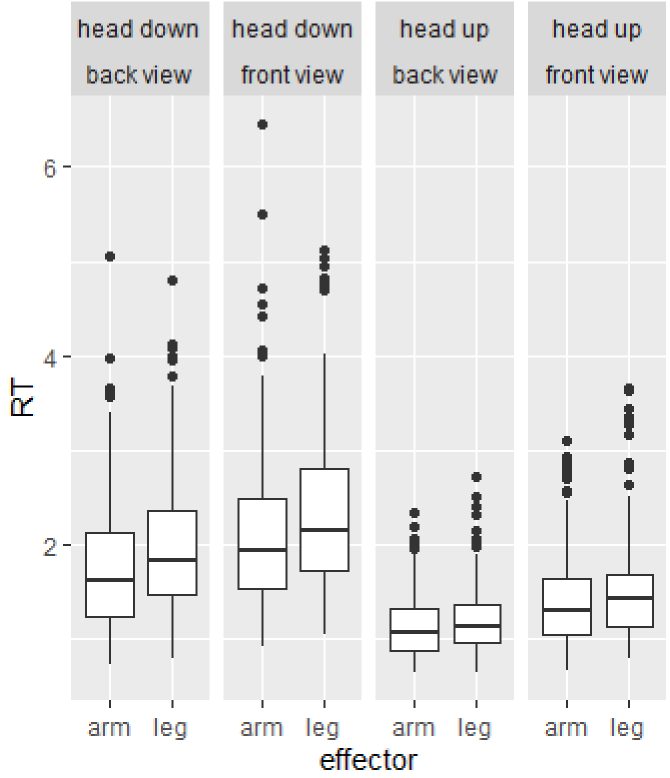

Analysis of error rates

A repeated measures ANOVA with the within-subject factors of perspective (front view, back view), rotation (head up, head down), and limb (arm, leg) was calculated on the error rates (percentage of errors). Boxplots of the error rates are shown in Figure S2.

The significant main effect of perspective, F (1, 152) = 24.6, $p < .001$, $\eta_p^2 = .14$, indicated significantly more errors in the front view (M±SD = 8.9±12.7 %) than the back view (M±SD = 6.4±10.5 %). The significant main effect of rotation, F (1, 152) = 63.9, $p < .001$, $\eta_p^2 = .3$, indicated significantly more errors in head down rotations (M±SD = 11.3±14.4 %) than head up rotations (M±SD = 4±6.5 %). The significant main effect of limb, F (1, 152) = 4.1, $p = .045$, $\eta_p^2 = .03$, indicated significantly more errors for leg items (M±SD = 8±11.9 %) than arm items (M±SD = 7.3±11.5 %). The interaction between rotation and limb was not significant, F (1, 152) = 1.9, $p = .176$, $\eta_p^2 = .01$. Further, all remaining interactions were not significant, $\eta_p^2 < .01$.

Figure S2. Boxplots of the error rates (ERR in %) depending on rotation (head down, head up), perspective (back view, front view), and limb (arm, leg) in Study 1.

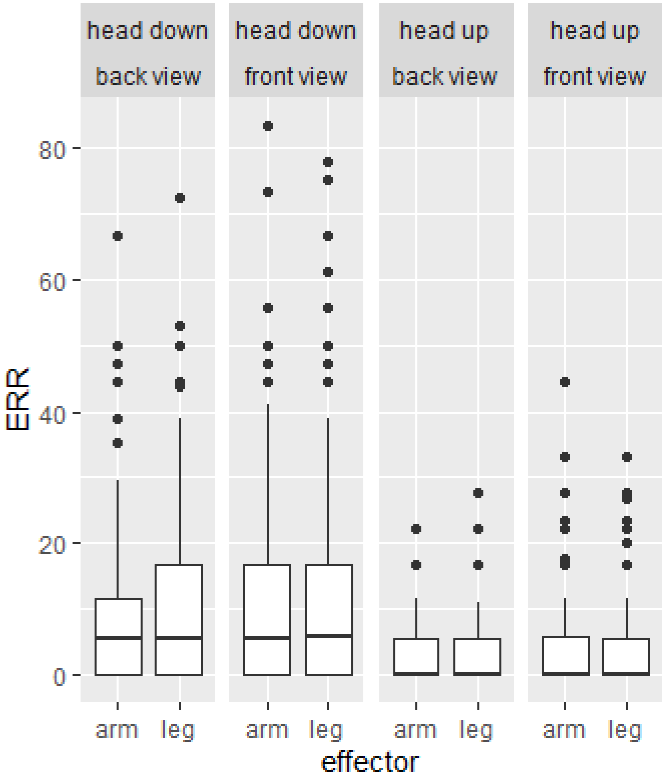

Correlations between response times and error rates

Figure S3 shows the correlations between error rates and response times. The right lower triangle indicates that response times correlated strongly between each other (*r* > .5). The left upper triangle indicated medium correlations between error rates (*r* > .3). Error rates and response times did not correlate with each other or showed small correlations (.2 < *r* < .2). Particularly, error rates in the back view and head up position that did not involve imagined rotations showed small negative correlations with the response times (-.3 < *r* < -.1). Scatterplots are shown in Figure S4.

Figure S3. Pearson correlations between error rates (ER in %) and response times (RT in s) depending on rotation (HD: head down, HU: head up), perspective (BV: back view, FV: front view), and limb (arm, leg) in Study 1.

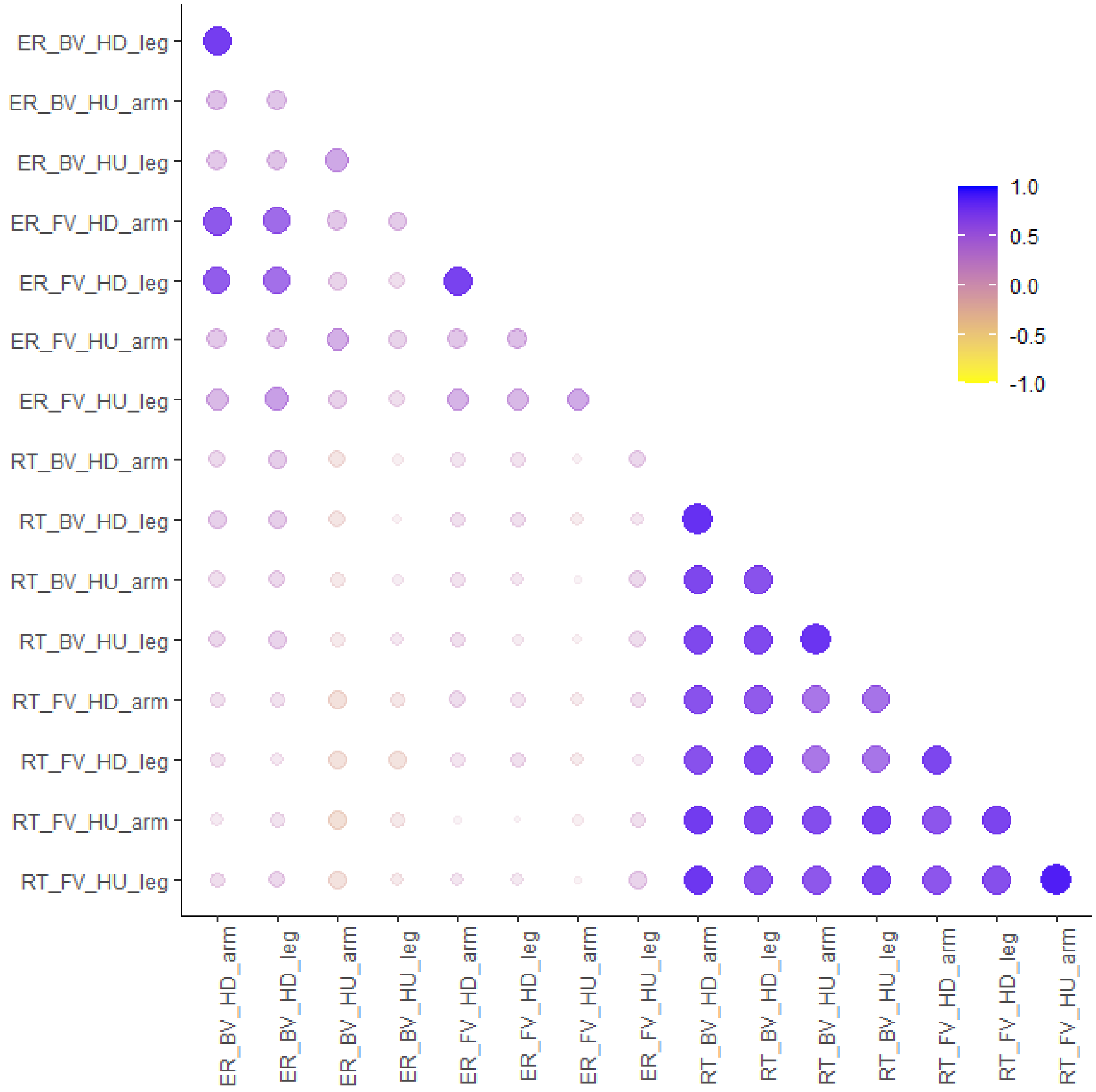

Figure S4. Scatter plots and Pearson correlations between error rates (ER in %) and response times (RT in s) depending on rotation (HD: head down, HU: head up) in Study 1.

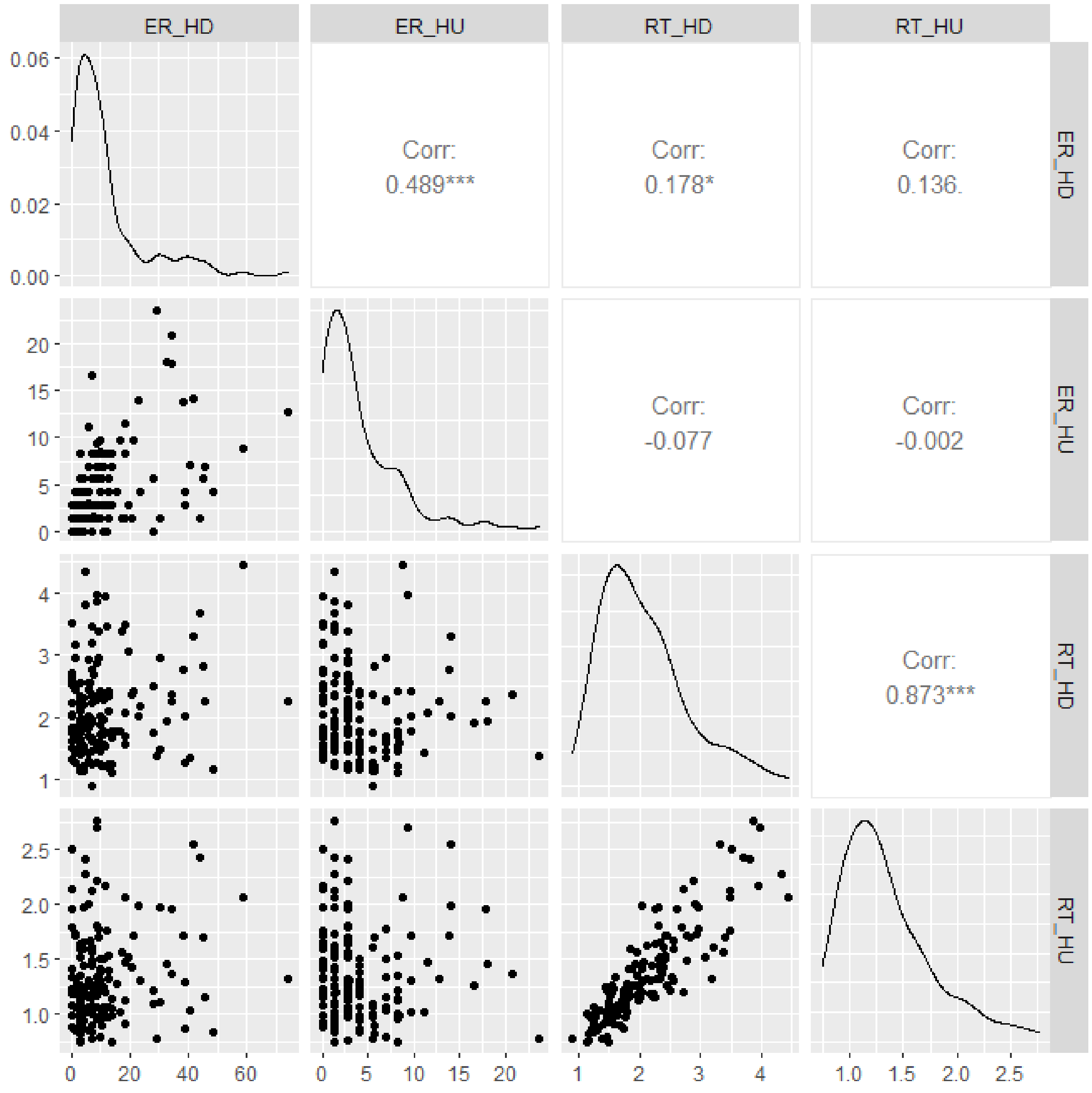

Correlations of age and imagery ability ratings with LISAS

To explore the effects of age and vividness of movement imagery ability (Roberts et al., 2008), we calculated a single LISAS that involves the mean of all conditions (perspective, rotation, limb, abstractness). Pearson correlations between LISAS and age, external visual imagery, internal visual imagery, and external visual imagery are shown in Table S1 for both studies. Note that variation in age was larger in Study 2 ($M \pm SD$ = 28.5±10.3 years) than in Study 1 ($M \pm SD$ = 21±4.5 years) which might explain the larger correlation, indicating better performance (lower LISAS scores) in younger participants than in older participants.

Table S1. *Pearson Correlations between age (in years), external visual imagery (EVI), internal visual imagery (IVI), kinesthetic visual imagery (KIN) and the linear-integrated speed-accuracy score (LISAS)*

	LISAS	
	Study 1	Study 2
Age	.16	.47
EVI	.07	.06
IVI	.06	.03
KIN	.11	.09

Learning in effects in Study 2

To explore learning effects in Study 2, a repeated measures ANOVA with the within-subject factor order (1, 2, 3, 4) was calculated on the LISAS. Boxplots of the LISAS are shown in Figure S5. The significant main effect of order, F (2.5, 304.6) = 28.7, p < .001, η_p^2 = .22, indicated a significant decrease of LISAS from the first to the second block (p = .023, d = .14), from the second to the third block (p < .001, d = .24), and from the third to the fourth block (p = .005, d = .12).

Figure S5. Boxplots of the linear integrated speed-accuracy scores (LISAS) depending on the order of the blocks in Study 2.

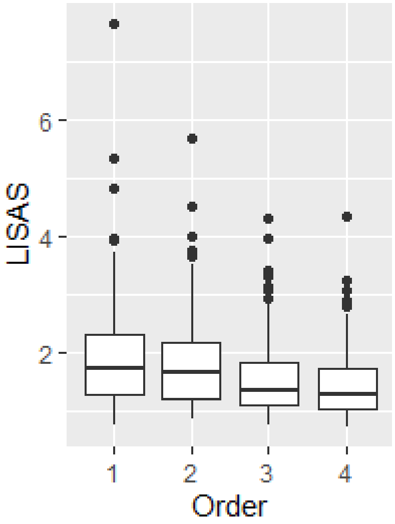

Effects of gender in Study 1 and Study 2

To explore effects of gender, a independent sample t-test was calculated on the LISAS. Boxplots of the LISAS in Study 1 are shown in Figure S6. In Study 1, the LISAS was significantly lower in male participants (*M*±*SD* = 1.5±0.5) than in females (*M*±*SD* = 1.9±0.7), t (42)= 3.8, $p < .001$, $d = 0.74$). Boxplots of the LISAS in Study 2 are shown in Figure S7. In Study 2, the LISAS did not significantly differ between male participants (*M*±*SD* = 1.4±0.6) and females (*M*±*SD* = 1.3±0.5), t (88.4)= 0.2, $p = .828$, $d = 0.04$). In both Study 1 and Study 2 the two gender groups reported here (female and male) were not balanced, and the proportion of females to males was substantially larger in Study 1.

Figure S6. Boxplots of the linear integrated speed-accuracy scores (LISAS) depending on gender in Study 1.

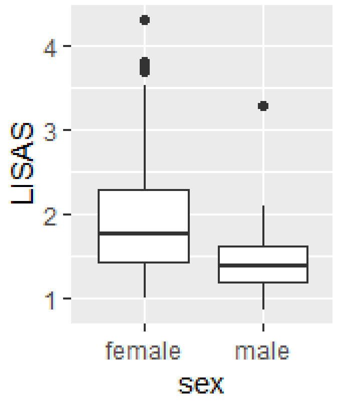

Figure S7. Boxplots of the linear integrated speed-accuracy scores (LISAS) depending on gender in Study 2.

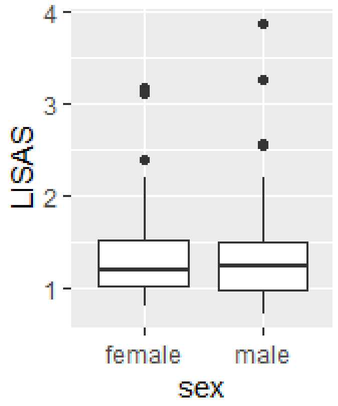

To explore interactions between sociodemographic variables (gender and age) and the variables in the mental body rotation task (rotation, perspectice, limb, and abstractness), we calculated explorative ANCOVAs. To prevent redundancies with the manuscript, we will only focus on significant main effects and interactions with the sociodemographic variabes in the following.

In Study 1, an ANCOVA with the between-subject factor of gender (female, male), the within-subject factors perspective (front view, back view), rotation (head-

up, head-down), and limb (arm, leg), and the covariate age was calculated on the LISAS. The significant main effect gender, F (1, 149) = 8.1, p = .005, η_p^2 = .05, indicated a significantly lower LISAS in male participants (M = 1.5) than in female participants (M = 1.94). The significant interaction between gender and rotation, F (1, 149) = 5.1, p = .026, η_p^2 = .03, indicated that the difference between head-up and head-down stimuli was significantly larger in female participants (M = 1) than in male participants (M = 0.68, p < .001).

The significant interaction between age and limb, F (1, 149) = 4.2, p = .043, η_p^2 = .03, was modified by the significant interaction between age, limb, and rotation, F (1, 149) = 5.6, p = .019, η_p^2 = .04. Post-hoc analyses indicated that age correlated significantly with head-down leg items (r = .19) and with head-up arm items (r = .21), but not with head-down arm items (r = .1) or head-up leg items (r = .14).

In Study 2, an ANCOVA with the between-subject factor of gender (female, male), the within-subject factors perspective (front view, back view), rotation (head-up, head-down), limb (arm, leg), and abstractness (realistic, abstract), and the covariate age was calculated on the LISAS. Boxplots of the LISAS are shown in Figure S8.

The significant interaction between gender and effector, F (1, 119) = 4.1, p = .044, η_p^2 = .03, did not reveal any significant post-hoc comparisons (see Figure S7).

Figure S8. Boxplots of the linear integrated speed-accuracy scores (LISAS) depending on gender (female, male) and limb (arm, leg) in Study 2.

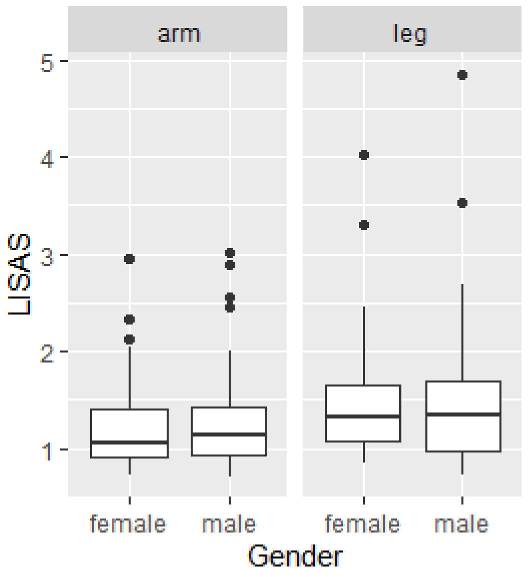

The significant main effect age,F (1, 119) = 36.2, p < .001, η_p^2 = .23, indicated a significant correlation between age and LISAS (r = .47). The interaction between age and limb, F (1, 119) = 20, p < .001, η_p^2 = .14, the interaction between age, limb and rotation, F (1, 119) = 4.9, p = .03, η_p^2 = .04, and the interaction between age, limb, rotation, and perspective, F (1, 119) = 5.2, p = .024, η_p^2 = .04, were significant. Fisher's z post-hoc comparisions indicated that age correlated significantly higher

with head-up back-view arm items (r = .6, z = 1.72, p = .043) and with head-up back-view leg items (r = .62, z = 2, p = .021) than with the other items (.34 < r < .434).

Significant interaction between age and abstractness, F (1, 119) = 7.3, p = .008, η_p^2 = .06, the interaction between age, abstractness, and limb, F (1, 119) = 10.5, p = .002, η_p^2 = .08, significant. The correlations (.4 < r < .48) did not significantly differ from each other (p_{min} = .217).
